# Influence of Tool Shape on Surface Quality of Monocrystalline Nickel Nanofabrication

**DOI:** 10.3390/molecules27030603

**Published:** 2022-01-18

**Authors:** Jie Ren, Haibao Yue, Guoxing Liang, Ming Lv

**Affiliations:** 1 State Key Laboratory of Precision Measuring Technology & Instruments, Centre of MicroNano Manufacturing Technology, Tianjin University, Tianjin 300072, China; 2 Department of Mechanical Engineering, Taiyuan Institute of Technology, Taiyuan 030008, China; 3 Technology Center, Taiyuan Heavy Industry Co., Ltd., Taiyuan 030024, China; yhbsmart@163.com; 4 Shanxi Key Laboratory of Precision machining, College of Mechanical Engineering, Taiyuan University of Technology, Taiyuan 030024, China; liangguoxing@tyut.edu.cn (G.L.); lvming@tyut.edu.cn (M.L.)

**Keywords:** monocrystalline nickel, molecular dynamics, nanofabrication, tool shape, surface roughness

## Abstract

In this paper, the influence of tool shape on the surface quality of monocrystalline nickel nanofabrication is studied. The research mainly adopts the method of molecular dynamics simulation, through the statistics of the atomic coordinates of the machined surface, then calculates the influence of different tool rake angles on the surface roughness of monocrystalline nickel. It is concluded that the surface roughness distribution is ‘W’ when the rake angle of the diamond tool changes from −45° to +45°. When analyzing the relationship between the tool shape and the processing temperature, it is found that when the clearance angle of the tool reaches a certain range, the clearance angle is further increased, and the temperature of the workpiece does not change during machining. Therefore, a large number of simulations were carried out, and it was concluded that there is a critical clearance angle, and the critical clearance angle of the tool in the research conditions is 8–10°.

## 1. Introduction

**Figure 1:**
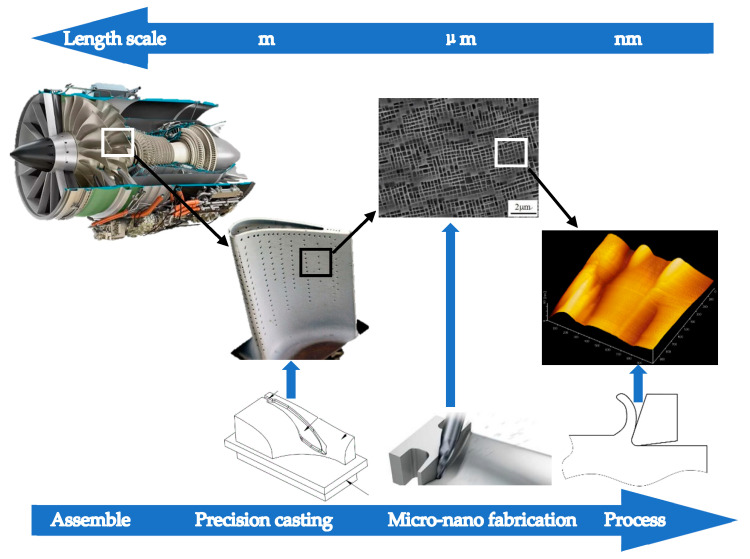
Ultra-precision parts of aero-engines. In the previous study of monocrystalline nickel grinding, it was found that the size of the abrasive grains had a significant influence of surface quality of monocrystalline nickel. The effect of abrasive particle diameter is essentially the reaction of an effective rake angle during processing. Thus, it can be concluded that the size and shape of the machining tool will affect the workpiece even when the size range is small to the nanometer level. Therefore, this study discusses the nanofabrication of tools with different rake angles, different clearance angles, and different tool edge radii, and their impact on the processing mechanism of monocrystalline nickel.

Monocrystalline nickel and its alloys are used as one of the materials for aero-engines. Due to the high-quality requirements of processing, many processings have been accurate to the nanometer level, as shown in Figure 1. The current nanofabrication technology mainly focuses on the research on different processing speeds, processing depths, and different crystal planes. Due to the reduction of machining allowance and the improvement of machining accuracy, the influence of the rake angle, clearance angle, and edge radius on the machining process and machining quality is significantly improved [1]. Research on the influence of the tool shape during the nanofabrication of monocrystalline nickel will help in improving the processing technology. The limitations of nanofabrication in experimental observations [2] make molecular dynamics a convenient and reliable method to research ultra-precision machining and nanofabrication [3,4].

In addition to the seminal work of Komanduri, Goel, and Markopoulos [5,6,7], the application of molecular dynamics in nanomaterials has been explored by several scholars. Papanikolaou et al. [8] studied the influence of the grain size of the workpiece on the nano-cutting process, and concluded that when cutting polycrystalline aluminum, the cutting force increases and friction coefficient decreases with the increase of grain size. Liu [9] et al. conducted molecular dynamics simulation on thermal vibration-assisted composite machining and found that temperature was proportional to the number of vacancy defects in the mixed machining process. Zhou et al. [10] established a molecular dynamics model for abrasive cutting, and dynamically tracked titanium atoms from the beginning of cutting to the end of cooling. The residual stress in abrasive cutting was studied, and the residual stress in the depth direction was mainly compressive stress, while the residual stress parallel to the cutting direction was mainly tensile stress.

With regard to the influence of tool shape on nano-machining, some scholars have also conducted research. Dai [11] et al. studied the nano-machining of single-crystal silicon by diamond tools, and discussed the influence of tool geometry on material deformation. Through the study of phase change, workpiece deformation, etc., the influence of tool shape on machining is revealed. The conclusion is that the size of the negative rake angle is proportional to the shear stress, and the positive rake angle and the smaller tool tip radius can help improve the workpiece surface smoothness after processing. Fang et al. [12] analyzed the relationship between the tool radius and the undeformed chip thickness (UCT) in the two cutting states of shear and extrusion. The results show that the influence of tool radius on the size effect of machining cannot be ignored, and the influence increases as the machining thickness decreases. Xu et al. [13] used molecular dynamics methods to reconstruct three different tool edges and studied the difference of the proposed parameters on the nano-machining process. In the article, the shape of the cutting edge of the typical symmetrical circular tool was changed. By establishing three asymmetric circular cutting edges and performing nano-cutting simulation on them, it was concluded that the new tool model can better characterize the cutting process, subsurface damage, cutting force, etc.

Some documents [14,15] also studied the influence of tool angle or shape on nanofabrication. However, most of the studies as mentioned above focused on qualitative trend analysis in one aspect of processing, such as the relationship between tool rake angle and shear force, and the relationship between tool radius and size effect. However, the quality of the machined surface, as an important standard for inspecting parts, has not been quantitatively studied. Therefore, this paper calculates the influence of different rake angles, different clearance angles, and different edge radii of the tool on the surface roughness of the workpiece. Besides, this paper defines the concept of critical relief angle by analyzing the temperature change after monocrystalline nickel processing, which provides a theoretical basis for actual processing.

## 2. Simulation Model

In this article, the molecular dynamics simulation model and analysis were established using the open source large-scale atomic and molecular parallel simulator (LAMMPS) [16], at the same time, OVITO [17] was used to visualize kinetic results and analyze data in a later stage.

**Figure 2:**
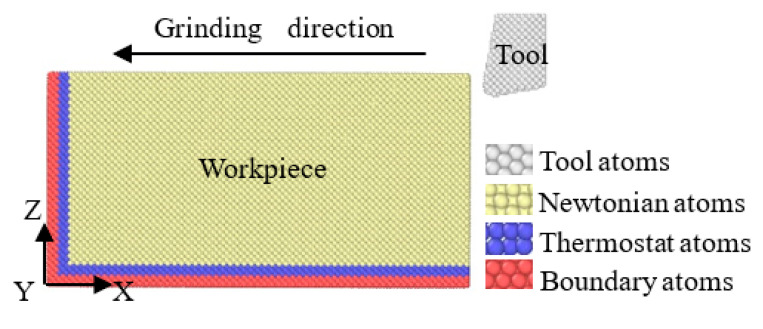
Molecular dynamics models of different tool shapes.

The molecular dynamics model used to study the nanofabrication of monocrystalline nickel with different tool shapes is shown in Figure 2. According to the lattice constant of monocrystalline nickel, the workpiece material is monocrystalline nickel, and due to periodic boundary conditions, the size is set to 21.2 × 10.6 × 10.6 nanometers, and the Y direction is the periodic boundary condition. Processing is simulated on the (100) plane. In order to be as close to the actual processing conditions as possible, different attributes are designed for the atoms in the workpiece. Boundary atoms are set up, which have a speed of 0 during processing to fix the workpiece. The thermostat atoms are kept at 293 K to simulate heat dissipation. Newtonian atoms are the key research part in processing by following Newton’s law. Table 1 lists the MD simulation conditions of nanomachining with different tool shapes.

## 3. Simulation Result and Analysis

### 3.1. Influence of Rake Angle on Chip Morphology

**Figure 3:**
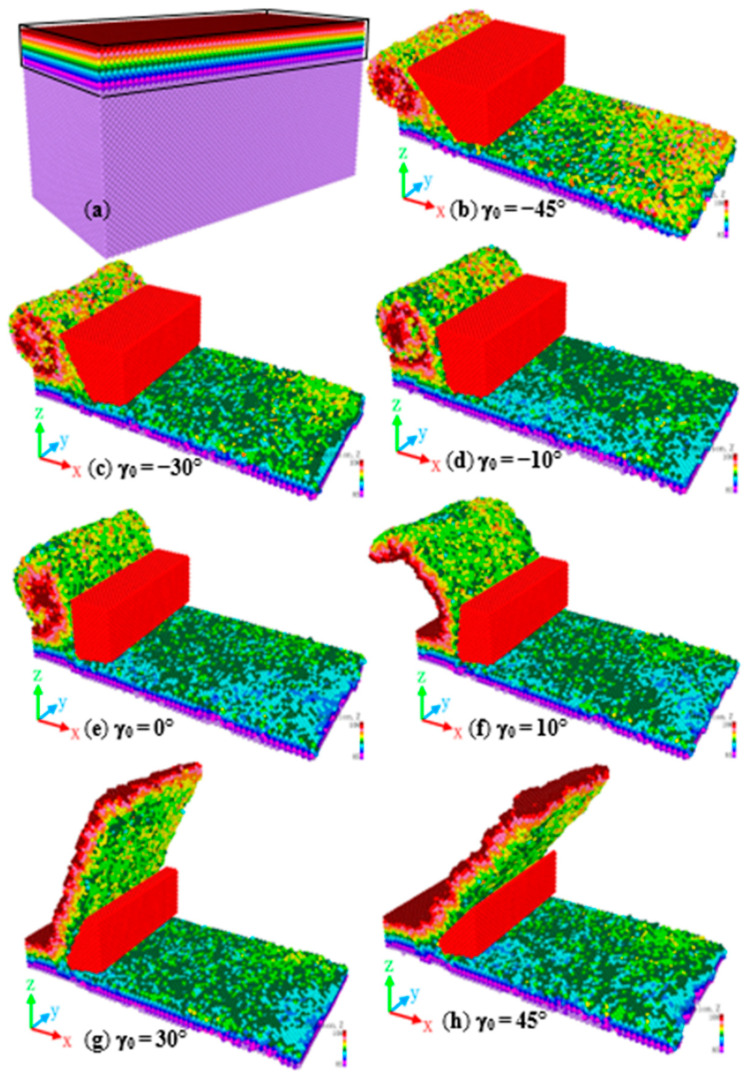
Chip morphology of monocrystalline nickel processed by different tool rake angles.

In order to more intuitively observe the purpose of chip formation in nanofabrication, the surface and subsurface atoms of the model are colored, and the initial color properties of the atoms in the workpiece are frozen, so that the atomic color remains unchanged during processing. These atoms are intercepted for observation, exhibited in Figure 3a. Figure 3b–h demonstrated the chips of nanofabrication monocrystalline nickel and the condition of the processed surface. At different rake angles when the machining depth is 1 nm, the machining speed is 200 m/s, the tool clearance angle *α*_0_ is 10°, at the same time, the tool edge radius *r* is 0.5 nm. As can be seen from the figure, when the rake angle of the tool is positive, the chips are strip-shaped, The processed surface atoms are mostly green. This is because the tool is relatively ‘sharp’ at this time, so the position of the shunting point of the tool is low, and most of the atoms in contact with the tool form chips, rather than being squeezed under the tool after being shunted. When the tool has a negative rake angle, the chips are piled up. This is because the formed chips move to the workpiece surface due to the extrusion of the tool rake face. When the tool has a negative rake angle and the and the value changes from −45° to −30°, to −10°, the number of yellow and orange atoms on the machined surface gradually increases. This is because the reduction of the rake angle makes the main processing method change from shearing to extrusion, and the position of the tool diversion point [20] during processing is improved. More atoms close to the surface are squeezed and left on the processed surface.

### 3.2. Effect of Rake Angle on Ra and Rz

The surface roughness of the processed parts is an essential standard for measuring the quality of processing, and it has an important effect on the use and life of the parts [21]. As so to research the connection between tool shape and surface roughness, the values of Ra and Rz were calculated, respectively.

According to the definition of surface roughness [22,23], the Ra value is

(1) $$\mathrm{Ra}=\frac{1}{l}\overset{1}{\underset{0}{\int }}\left|y\right|\mathrm{dx}$$

The formula can be approximated as

(2) $$\mathrm{Ra}\approx \frac{1}{n}\overset{n}{\underset{i=1}{\sum }}\left|y\right|$$

where |y| represents the offset of the contour line, that is, the contour point, the distance from the reference line in the measuring direction. L is the sampling length, and n is the number of test points. The baseline is the contour centerline used to evaluate the surface roughness parameters. There are two types of reference baselines: the least-squares centerline of the contour; the sum of the squares of the distance, which is between each point on the contour line; and the least-squares centerline, which is the smallest, within sample length. The arithmetic means centerline of the contour: the area of the upper and lower contours on the centerline is equal. Theoretically, the least-squares centerline is an ideal baseline, but it is not easy to obtain in practical applications. Therefore, the arithmetic mean centerline of the contour is generally used instead, and a straight line with an approximate position can be used for measurement.

Set straight line as

y = ax + b (3)

Substituting Formula (3) into Formula (2), we get

(4) $$\mathrm{Ra}\approx \frac{1}{n}\overset{n}{\underset{i=1}{\sum }}\left|y_{i}-{\mathrm{ax}}_{i}-b\right|$$

In Equations (3) and (4), a is the slope, b is a constant, and n is the number of test points taken.

**Figure 4:**
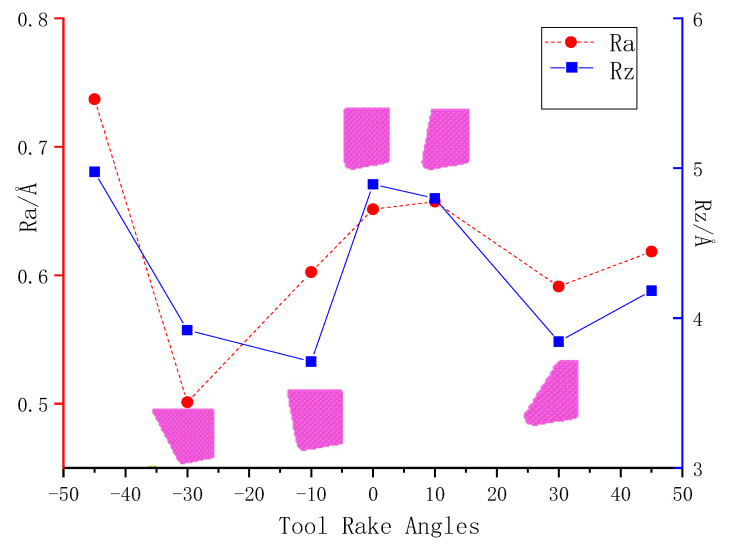
Workpiece surface roughness after machining with different rake angles.

Count the atomic coordinates of the surface, get the values of a and b in Formula (4) by fitting a straight line, then the value of Ra under different tool rake angles is obtained through a large number of calculations. The unit is Å, as shown in Figure 4.

The Figure 4 shows that when the rake angle of the tool is positive and the value changes from 10° to 30°, to 45°, because the tool is ‘sharp’, the processed surface atoms are mainly subjected to shearing, so fewer atoms are moving with the tool to the processing direction. It can be seen from the comparison of Figure 3e–h that when the current angle is 30°, the distribution of green atoms on the processed surface is more uniform, indicating that the surface layer atoms move less, and most of them remain at the original height position, and the surface roughness value is smaller.

When the machining tool has a negative rake angle, and the value changes from −45° to −30°, to −10°, comparing illustration in Figure 3b–d, while the rake angle is an obtuse angle, the surface will be more ‘flat’ due to the extrusion of the chip by the tool, and the surface quality has been improved to a certain extent. A very complex stress field and strain field will be generated inside the material during the cutting process of a negative rake angle tool, which will cause material retention on the rake face of the tool. The retention phenomenon will lead to uneven distribution of processed materials, and significantly reduce the mechanical properties and surface quality of the processed material. As a result, when the rake angle is −45°, the surface roughness increases sharply.

### 3.3. Influence of Tool Shape on Cutting Force

**Figure 5:**
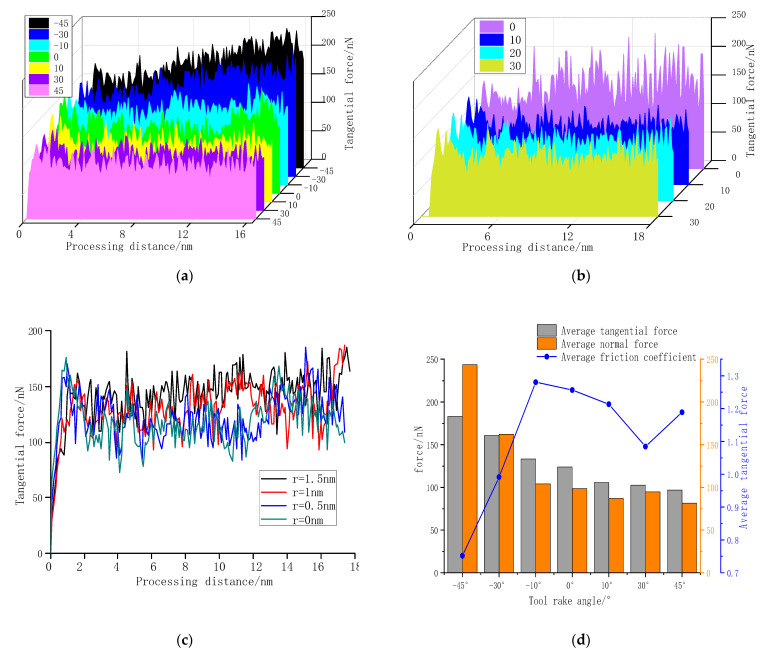
Change in tangential force during processing. (**a**) The tool clearance angle and the edge radius are the same, when the tool rake angle is different. (**b**) The tool rake angle and the edge radius are the same, and the tool clearance angle is different. (**c**) The front and clearance angles of the tool are the same, and the edge radius are different. (**d**) The relationship between friction coefficient and rake angle.

Figure 5 performance the change of tangential force under different tool shapes. According to Figure 5a, when the tool has a constant edge radius and clearance angle, the cutting force decreases as tool rake angle increases. As metal cutting is the result of cutting and squeezing the metal material by the tool: the larger the rake angle, the greater the shearing effect on the workpiece atoms; the smaller the rake angle, the greater the squeezing effect on the workpiece atoms. When the tool has a negative rake angle, interatomic force and the tangential force increase due to the squeezing of the chip atoms and the tool rake face. When the tool angle increases, the number of atoms of the workpiece in contact with the tool decrease, the plastic deformation range and degree of deformation decrease, and the friction of the chips along the rake face also decreases [24], thereby significantly reducing the cutting force. At the same time, comparing the shape of the chip and the contact area with the tool in Figure 3, it can also be found that when the tool rake angle is positive, the chips are striped, and the contact area between the chip atoms and the tool is smaller than when the tool rake angle is negative, the number of chip atoms are also smaller, so the chip atoms have less force on the tool, and the cutting force is also smaller. However, when the rake angle is increased from 30° to 45°, it can be seen from Figure 4 that the tangential force does not change much, while the excessive rake angle will cause the tool to become thinner and reduce the strength. Therefore, the appropriate tool rake angle should be selected during processing.

Figure 5b demonstrated the change of tangential force on the move of nanofabrication monocrystalline nickel with different tool clearance angle while the tool rake angle and edge radius are fixed. as shown in the graph that when the tool clearance angle is increased from 0° to 10°, the tangential force is significantly reduced. The workpiece atoms will produce elastic deformation and plastic deformation under the action of the tool, and some of the machined surface atoms will recover in the Z direction due to the elastic deformation after the tool passes. These atoms create a certain amount of friction with the clearance angle of the tool. This is due to the increase of the tool clearance angle, which reduces the friction between the flank face and the transition surface during the cutting process, thereby reducing the tangential force. When the tool clearance angle increases from 10° to 30°, the tangential force basically does not change. This is because after the clearance angle exceeds a particular value, the flank face cannot touch the workpiece. Therefore, as long as the clearance angle is larger than this value, the tangential forces are basically the same.

According to Figure 5c, when the cutting depth is 1 nm, and the cutting speed is 200 m/s, when the radius of the tool’s blunt circle is 0–1.5 nm, the tangential force does not change much.

The friction coefficient (μ) is defined as the tangential force (Ft) divided by the normal force (Fn). According to Figure 5d when the rake angle of the tool is −45°, since the chips are curled and accumulated on the rake face, the force of the tool on the chips is mainly the normal force, so the friction coefficient is small. When the rake angle of the tool increases, the chips change from a curled shape to a strip shape, and the atoms directly acted on by the tool decrease, so both the tangential force and the normal force are reduced, and the friction coefficient changes significantly. Therefore, the friction coefficient has a great relationship with the shape of the chip, and the rake angle of the tool directly affects the shape of the chip, so the rake angle of the tool has a great influence on the friction coefficient.

### 3.4. Influence of Tool Shape on Temperature

**Figure 6:**
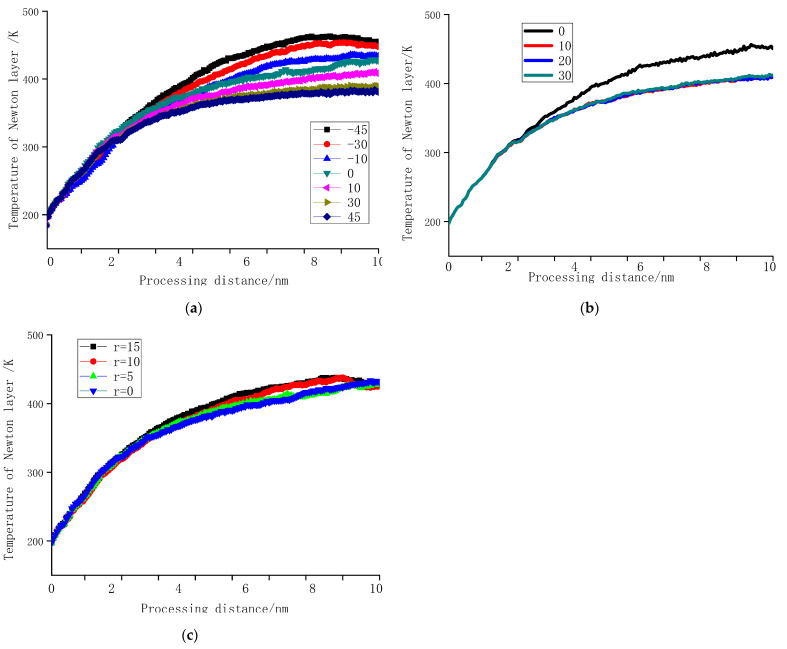
Temperature change of the workpiece Newton layer during machining. (**a**) The tool clearance angle *α*_0_ = 10° and the edge radius *r* = 0.5 nm, when the tool rake angle is different. (**b**) The tool rake angle *γ*_0_ = 10° and the edge radius *r* = 0.5 nm, and the tool clearance angle is different. (**c**) The front and clearance angles of the tool are the same, while *α*_0_ = 10° and *γ*_0_ = 10°, the edge radius is different.

Figure 6 shows the temperature change of the workpiece in Newtonian layer during processing. By comparing Figure 4, it can be seen that the changing trend of temperature is the same as that of cutting force. Because classical molecular dynamics ignores the contribution of electrons to temperature, and due to the model setting, there is a certain distance between the constant temperature layer and the processing surface, the temperature control slightly deviates from reality. Therefore, this study does not discuss the value of temperature, and only compares settings with each other under the same deviation.

As shown in Figure 6a: when the tool rake angle decreases, the temperature of the workpiece improves. This is because the negative rake angle in the processing process—due to the squeezing effect—makes the workpiece atom’s force area larger and force time longer, generating more heat, so the temperature is higher. The temperature of the workpiece is directly related to the cutting force. It can be seen from Figure 6c that under the conditions of this study, the tool radius has little effect on temperature.

**Figure 7:**
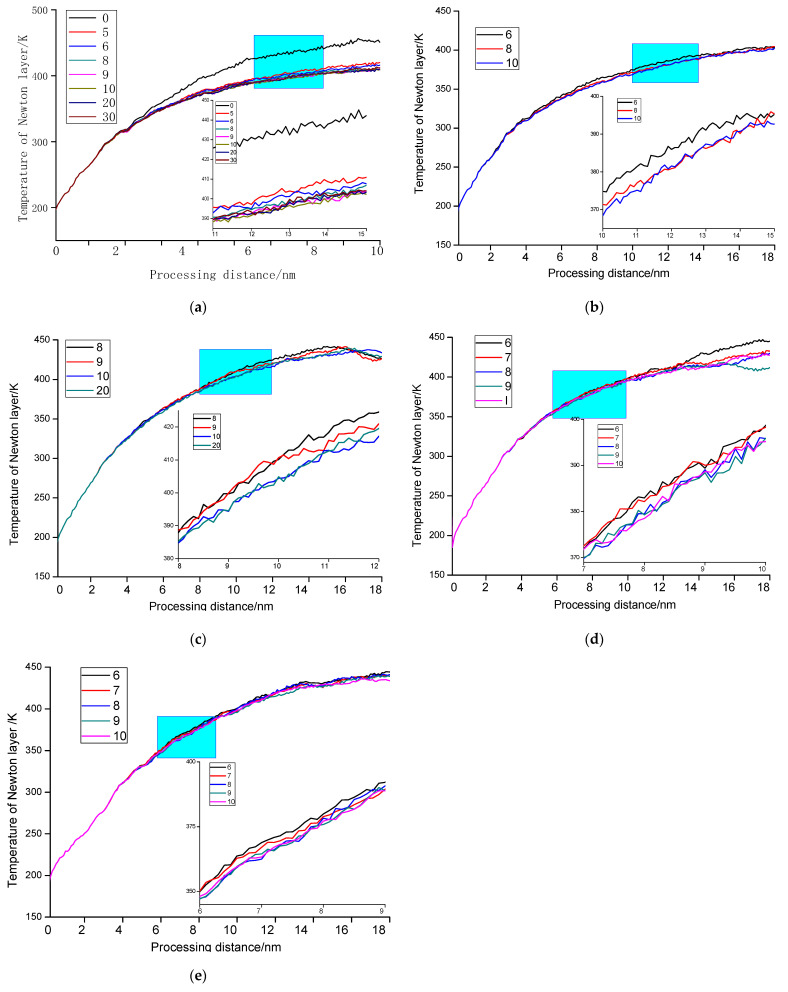
Effect of different tool clearance angles on workpiece temperature with a machining depth of 1 nm and a machining speed of 200 m/s (**a**) *r* = 0.5 nm, *γ*_0_ = 10°; (**b**) *r* = 0 nm, *γ*_0_ = 10°; (**c**) *r* = 1.5 nm, *γ*_0_ = 10°; (**d**) *r* = 0.5 nm, *γ*_0_ = 0°; (**e**) *r* = 0.5 nm, *γ*_0_ = −10°.

Figure 6b reveals the temperature change of the workpiece when nanofabrication with different tool clearance angle. While the cutting depth is 1 nm, the cutting speed is 200 m/s, the tool rake angle *α*_0_ is 10°, and the tool edge radius *r* is 0.5 nm. When the tool clearance angle is 10°, 20°, and 30°, the temperature curve is basically the same. Therefore, it can be considered that when the tool clearance angle is greater than a particular value, the machined surface is not affected by the friction of the relief surface, and the temperature curve is the same. In order to determine the range of this value, multiple sets of processing simulations were carried out, and the drawn temperature curve is shown in Figure 7.

It can be seen from Figure 7 that when the clearance angle increases to a critical angle, the temperature no longer changes with the increase of the clearance angle. However, the increase of the clearance angle will make the tool thinner and reduce the strength of the tool. Therefore, it can be concluded that there is a critical angle in the clearance angle of monocrystalline nickel during nanofabrication. The value of the tool’s back angle is within the critical angle range, which will have less friction and offer higher strength to the tool.

As can be seen from Table 2, when the cutting depth is 1 nm and the cutting speed is 200 m/s, The critical range of tool clearance angle is 8–10°.

### 3.5. Influence of the Rake Angle on the Chip Lattice

**Figure 8:**
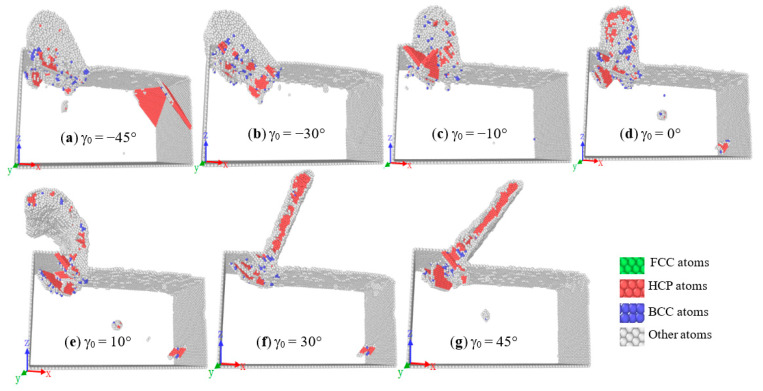
CAN analysis.

Figure 8 shows the common neighbor analysis (CNA) of the workpiece when the different tool rake angle is processed and the clearance angle and radius are fixed. For easy observation, the FCC atoms in the workpiece are removed. It can be seen from the figure that when the tool rake angle changes from positive to negative, the friction between the rake face and the chips increases, which increases the force on the chips, and more FCC atomic bonds are destroyed.

**Figure 9:**
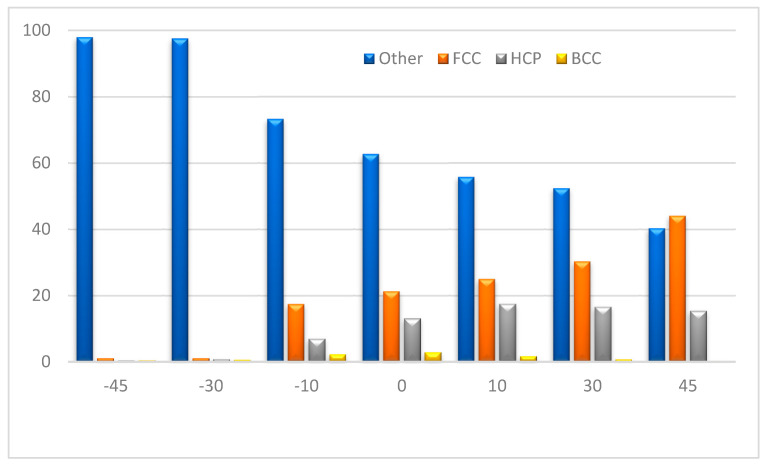

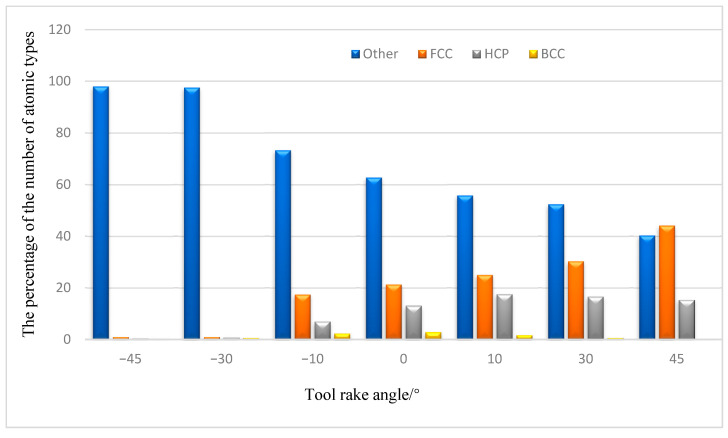
Percentage of different crystal structures in the wear chips.

Figure 9 shows the percentage of different crystal structures in the chip. It can be seen intuitively that as the rake angle of the tool decreases, the percentage of FCC atoms decreases, and the number of other atoms increases. Other atoms are mainly amorphous atoms and surface atoms. This is because when the tool negative rake angle is large, the friction between the tool and the chip increases, the cutting force and temperature increase, and most of the energy produced in processing is converted into heat. The remaining small part of the energy is stored by the workpiece atoms and becomes their internal energy [25], which includes new surface generation energy and the energy required to produce defects in the material, such as dislocations, vacancies, and gaps [26]. The arrangement and number of defects in the workpiece together determine the change of its stored internal energy. In addition, when some materials such as silicon are processed, a phase change occurs, from a single crystal phase to an amorphous or polycrystalline phase [27]. The internal energy of the chips increases, and the destruction of the atomic structure increases, which increases the number of other atoms in the chips. Whether the other atoms are amorphous nickel needs to be further verified by experiments.

## 4. Conclusions

1. When the rest of the machining conditions remain unchanged and the rake angle of the tool changes between −45° and +45°, the surface roughness value of the processed workpiece fluctuates in a ‘W’ curve through statistical calculation. The better range of surface roughness appears around the tool rake angles of +30° and −30°.
2. Cutting force and temperature is more sensitive to the change of the tool rake angle, while the edge radius of the tool has little effect on both.
3. In the case of a fixed tool rake angle and edge radius of the tool, the value of the tool clearance angle is changed by one degree. Through a large number of simulation comparisons, it is concluded that there is a critical area in the tool clearance angle, and the tool clearance angle does not work on the machined surface after it exceeds the critical area. Furthermore, it is concluded that when the single crystal nickel nano-processing depth is 1 nm, the critical clearance angle area is 8–10°.

## Figures and Tables

**Table 1:** Set-up conditions of grinding monocrystalline nickel molecular dynamic simulation.

Factor	Workpiece	Grinding Grain
Material type Potential used Dimension	Monocrystalline nickel Ni-Ni: EAM [18], Ni-C: Morse [19] 21.2 × 10.6 × 10.6 nm	Diamond 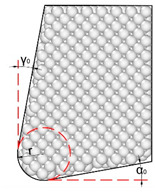
Atom number	225121	
Initial temperature Crystal orientation Grinding speed Depth of grind	293 K x-[100], y-[10], z-[1] 200 m/s 1 nm	
Grinding length Timestep	0~18 nm 1 fs	
Tool rake angle	−45°/−30°/−10°/0°/10°/30°/45° The average *α*_0_ = 0°	
Tool clearance angle	0°/10°/20°/30° The average *γ*_0_ = 15°	
Tool edge radius	0/0.5/1/1.5 nm	

**Table 2:** Clearance angle critical value statistics.

Tool Rake Angle *γ*_0_	Tool Edge Radius *r*	Critical Value of Clearance Angles *α*_0_
10°	0.5 nm	9°
10°	0 nm	9°
10°	1.5 nm	10°
0°	0.5 nm	8°
−10°	0.5 nm	8°

## Data Availability

The raw/processed data required to reproduce these findings cannot be shared at this time as the data also forms part of an ongoing study.

## Sample Availability

Samples of the compounds are available from the authors.
